# Factors associated with health intentions and behaviour among health checkup participants in Japan

**DOI:** 10.1038/s41598-021-99303-y

**Published:** 2021-10-05

**Authors:** Takayuki Otsuka, Tsuneo Konta, Ri Sho, Tsukasa Osaki, Masayoshi Souri, Natsuko Suzuki, Takamasa Kayama, Yoshiyuki Ueno

**Affiliations:** 1 Department of Public Health and Hygiene, Yamagata University Graduate School of Medical Science, Yamagata, Japan; 2 Institute for Promotion of Medical Science Research, Yamagata University Faculty of Medicine, Yamagata, Japan

**Keywords:** Epidemiology, Population screening

## Abstract

Health intentions and behaviours are essential for improving the health of individuals and society. This study used cross-sectional data from 20,155 health checkup participants in the Yamagata study to identify factors associated with health intentions and behaviours. Information regarding the current level of health intentions and behaviours was collected using a baseline survey questionnaire. Participants were categorised into three groups: having no intention (no intention), having intentions to improve but not acting on them (intention), and already active (action). The associations between background factors and the presence/absence of health intentions and behaviours were assessed using logistic regression analysis. Of the participants, 35.4%, 37.7%, and 26.9% belonged to the no intention, intention, and action groups, respectively. Multivariate analysis revealed that the factors associated with health intentions were being young, being female, longer duration of education, higher body mass index and abdominal circumference, diabetes, and dyslipidaemia. The factors associated with health behaviours were being older and male, not consuming alcohol, not smoking, performing daily exercise, and having diabetes. These results indicate that health guidance considering background factors, including age, gender, education, and comorbidities, may be useful for effectively promoting health intentions and health behaviours in the Japanese population.

## Introduction

Health intentions and behaviours are essential for improving the health of individuals and society. To improve the health status of society as a whole, it is crucial to improve individual health status. To achieve this goal, Japan implemented a national health promotion campaign called the second term of the National Health Promotion Movement in the twenty-first century (Health Japan 21 [the second term]), in 2013 ^1^. In this campaign, various health promotion activities, such as health classes and other educational programs, are being conducted to raise health awareness in the general population. However, an interim evaluation in 2019 revealed no increase in the number of citizens who are actively involved in health promotion activities ^2^. This indicates that the previous broad uniform approach for the general population was not effective. Therefore, to increase the number of people engaged in health promotion activities, it is necessary to identify the factors that promote and hinder health behaviours and implement new initiatives in response to these factors.

To actively pursue a state of health, individuals must have the intention to become healthy, and perform behaviours to act on that intention ^3^. “Health behaviours” are defined as actions taken by individuals that affect health or mortality ^4^. Health behaviours cover a wide range of areas, including diet, exercise, sleep, smoking, drinking, dental hygiene, and medical checkups ^5–12^. Before engaging in health behaviours, an individual is expected to have the intention to change health-related behaviours (referred to as “health intentions” here). However, various barriers affect human cognition, including present orientation bias. Therefore, even if people have health intentions, it is not necessarily easy to act on them.

Previous studies in Europe and the United States have shown that obtaining and understanding information about diseases leads to the development of health intentions and behaviours ^13^. However, such a process is strongly affected by environmental factors. Health-related environmental factors may differ depending on physical, psychological, and social conditions, including region, age, gender, ethnicity, and cultural background ^14–22^. Therefore, it is necessary to clarify the factors affecting health promotion among the Japanese population. Previous studies in Japan reported that approximately 50% of the Japanese population try to exercise and change their lifestyle habits to stay healthy ^23^. The number of people in their 50s and older taking action to improve their lifestyle is increasing ^24^. Health-conscious people are likely to participate in health centre programs, such as health classes and checkups ^25^. However, the factors directly related to health intentions and behaviours in a community have not yet been examined thoroughly.

To address this issue, this cross-sectional study examined the factors associated with health intentions and behaviours using data from health checkup participants from the Yamagata study.

## Results

In this study, data from 20,115 participants with a mean age of 62.8 years were used. The demographic characteristics of the study participants were as follows: male, 42.0%; living alone, 7.4%; habitual alcohol consumption, 46.5%; current smoking, 13.0%; history of hypertension, 37.3%; history of diabetes, 9.1%; history of dyslipidaemia, 18.9%; history of cancer, 5.1%; history of angina/myocardial infarction, 3.7%; and history of stroke, 2.6%. Regarding duration of education, 15.6% of participants received < 9 years of education, 53.3% received 10–12 years of education, and 31.2% received 13 or more years of education. The mean strolling jogging (SJ) value was 22.6 Mets × h/week. Each participant was categorised into the no intention group (7135 participants; 35.4%), the intention group (7608 participants; 37.7%) or the action group (5412 participants; 26.9%) (Table 1).

**Table 1:** Characteristics of study participants.

	Total N = 20,155	No intention N = 7135	Intention N = 7608	Action N = 5412	P-value
Age (year)	62.8 ± 8.4	63.7 ± 8.2	61.1 ± 8.8	64.2 ± 7.5	< 0.01
**Age category (year)**					
39–49	1915 (9.5%)	574 (8.0%)	997 (13.1%)	344 (6.4%)	< 0.01
50–59	3413 (16.9%)	1125 (15.8%)	1578 (20.7%)	710 (13.1%)	
60–69	10,120 (50.2%)	3451 (48.4%)	3726 (49.0%)	2943 (54.4%)	
70–74	4707 (23.4%)	1985 (27.8%)	1307 (17.2%)	1415 (26.2%)	
Male (%)	8465 (42.0%)	3440 (48.2%)	2883 (37.2%)	2192 (40.5%)	< 0.01
**Education period (year)**					
< 9	2849 (15.6%)	1262 (19.8%)	866 (12.5%)	721 (14.3%)	< 0.01
10–12	9754 (53.3%)	3404 (53.4%)	3723 (53.9%)	2627 (52.2%)	
> 13	5710 (31.2%)	1707 (26.8%)	2316 (33.5%)	1687 (33.5%)	
**Living alone (%)**					
Yes	1490 (7.4%)	450 (6.3%)	559 (7.4%)	481 (8.9%)	< 0.01
No	18,665 (92.6%)	6685 (93.7%)	7049 (92.7%)	4931 (91.1%)	
Body mass index (kg/m^2^)	23.0 ± 3.22	22.6 ± 3.02	23.4 ± 3.41	23.1 ± 3.14	< 0.01
Abdominal circumference (cm)	82.5 ± 8.8	81.2 ± 8.4	83.2 ± 9.1	82.8 ± 8.5	< 0.01
**Alcohol consumption (%)**					
Every day	4817 (24.0%)	2052 (28.8%)	1733 (22.9%)	1032 (24.1%)	< 0.01
Sometimes	4526 (22.5%)	1432 (20.1%)	1792 (23.6%)	1302 (24.1%)	
Never	10,755 (53.5%)	3638 (51.1%)	4057 (53.5%)	3060 (56.7%)	
**Smoking (%)**					
Current	2063 (13.0%)	1130 (15.9%)	1024 (13.6%)	449 (8.3%)	< 0.01
None	13,035 (65.0%)	4313 (60.6%)	5047 (66.8%)	3675 (68.3%)	
Quit	4425 (22.1%)	1679 (23.6%)	1486 (19.7%)	1260 (23.4%)	
**History of hypertension (%)**					
Yes	5768 (37.3%)	1902 (35.9%)	2190 (37.3%)	1676 (39.0%)	< 0.01
No	9705 (62.7%)	3397 (64.1%)	3689 (62.8%)	2619 (61.0%)	
**History of diabetes (%)**					
Yes	1346 (9.1%)	312 (6.2%)	463 (8.2%)	571 (13.9%)	< 0.01
No	13,476 (90.9%)	4754 (93.8%)	5190 (91.8%)	3532 (86.1%)	
**History of dyslipidemia (%)**					
Yes	2147 (18.9%)	666 (13.3%)	1167 (20.7%)	944 (23.2%)	< 0.01
No	11,937 (81.1%)	4342 (86.7%)	4475 (79.3%)	3120 (76.8%)	
**History of cancer**					
Yes	1033 (5.1%)	337 (4.7%)	373 (4.9%)	323 (6.0%)	< 0.01
No	19,122 (94.9%)	6798 (95.3%)	7235 (95.1%)	5089 (94.0%)	
**History of angina/myocardial infarction**					
Yes	678 (3.7%)	231 (3.6%)	253 (3.6%)	194 (3.8%)	0.80
No	17,801 (96.3%)	6201 (96.4%)	6711 (96.4%)	4889 (96.2%)	
**History of stroke**					
Yes	474 (2.6%)	147 (2.3%)	167 (2.4%)	160 (3.1%)	< 0.01
No	18,019 (97.4%)	6288 (97.7%)	6801 (97.6%)	4930 (96.9%)	
SJ-value(MetS × h/week)	22.6 ± 29.1	22.3 ± 30.4	17.9 ± 26.6	29.7 ± 29.3	< 0.01
**Exercise habits**					
Mild	4443 (35.6%)	1713 (39.7%)	2127 (44.2%)	603 (17.9%)	< 0.01
Moderate	3930 (31.5%)	1214 (28.2%)	1478 (30.7%)	1238 (36.8%)	
Intense	4122 (33.0%)	1385 (32.1%)	1213 (25.2%)	1524 (45.3%)	

Mean ± standard deviation.

**Figure 1:**
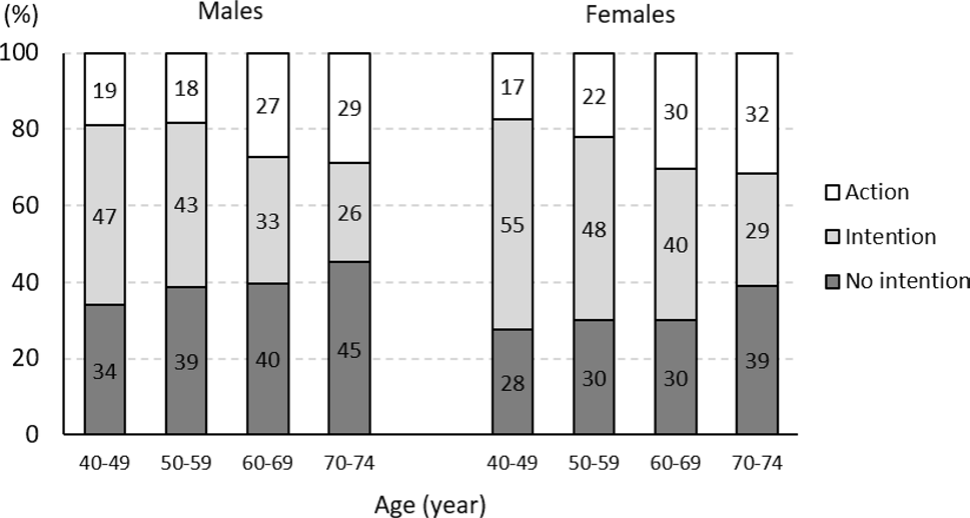
The prevalence of no intention, intention and action groups by gender and age.

Comparisons among the three groups revealed significant differences in characteristics, including age, gender, duration of education, living alone, alcohol consumption, current smoking, history of comorbidities, and exercise habits (Table 1). The no intention group contained the highest prevalence of males, individuals with a duration of education of < 9 years, habitual alcohol consumption, and current smoking. The intention group had the lowest mean age and the highest prevalence of females. The action group had the highest mean age and the highest prevalence of living alone, hypertension, diabetes, dyslipidaemia, cancer, and stroke. The gender- and age-stratified distribution of health intentions and behaviours revealed that the highest prevalence of being classified into the no intention, intention, and action groups was observed in 70–74-year-old males (45%), 40–49-year-old females (55%), and 70–74-year-old females (32%), respectively (Fig. 1).

Next, we examined the factors associated with being classified into the intention and no intention groups. The univariate analysis revealed that being young, being female, having a longer duration of education, living alone, having a high body mass index (BMI) and abdominal circumference, no alcohol consumption, no smoking, less daily exercise, diabetes, and dyslipidaemia were significantly associated with health intentions. The multivariate analysis revealed that being young, being female, having a longer duration of education, having a high BMI and abdominal circumference, less exercise habit, diabetes, and dyslipidaemia were independently associated with health intentions (Table 2).

**Table 2:** Factors associated with intention vs. no intention: logistic regression analysis.

	Univariate analysis		Multivariate analysis	
	OR (95% CI)	P-value	OR (95% CI)	P-value
Age (per 1-year increase)	0.97 (0.96–0.97)	< 0.01	0.96 (0.96–0.97)	< 0.01
Male gender (vs. female)	0.64 (0.60–0.68)	< 0.01	0.60 (0.53–0.67)	< 0.01
**Education period**				
10–12 years (vs. ≤ 9 years)	1.59 (1.44–1.76)	< 0.01	1.34 (1.13–1.58)	< 0.01
> 13 years (vs. ≤ 9 years)	1.98 (1.78–2.20)	< 0.01	1.63 (1.36–1.94)	< 0.01
Living alone	1.18 (1.04–1.34)	0.01	1.12 (0.93–1.36)	0.22
BMI (per 1 SD increase)	1.31 (1.27–1.35)	< 0.01	1.18 (1.07–1.30)	< 0.01
Abdominal circumference (per 1 SD increase)	1.30 (1.26–1.35)	< 0.01	1.28 (1.16–1.41)	< 0.01
Alcohol consumption	0.91 (0.85–0.97)	< 0.01	1.05 (0.94–1.17)	0.40
Current smoking	0.83 (0.76–0.91)	< 0.01	1.02 (0.88–1.18)	0.84
History of hypertension	1.06 (0.98–1.15)	0.14	1.08 (0.96–1.22)	0.17
History of diabetes	1.36 (1.17–1.58)	< 0.01	1.44 (1.16–1.80)	< 0.01
History of dyslipidemia	1.70 (1.53–1.89)	< 0.01	1.56 (1.35–1.80)	< 0.01
History of cancer	1.04 (0.89–1.21)	0.61	1.14 (0.91–1.42)	0.26
History of angina/myocardial infarction	1.01 (0.84–1.21)	0.90	1.04 (0.78–1.36)	0.81
History of stroke	1.05 (0.84–1.32)	0.67	1.08 (0.78–1.51)	0.63
Exercise moderate (vs. mild)	0.98 (0.89–1.08)	0.70	1.08 (0.96–1.22)	0.18
intense (vs. mild)	0.71 (0.64–0.78)	< 0.01	0.86 (0.76–0.98)	0.02

*OR* odds ratio, *CI* confidence interval, *BMI* body mass index, *SD* standard deviation.

We then examined the factors associated with the action and intention groups. The univariate analysis revealed that being older, being male, having a shorter duration of education, living alone, having a low BMI and abdominal circumference, no alcohol consumption, no smoking, daily exercise, diabetes, dyslipidaemia, cancer, and stroke were significantly associated with health behaviours. The multivariate analysis revealed that being older, being male, having a low BMI, no alcohol consumption, no smoking, daily exercise, and diabetes were independently associated with health behaviours (Table 3).

**Table 3:** Factors associated with action vs. intention: logistic regression analysis.

	Univariate analysis		Multivariate analysis	
	OR (95% CI)	P-value	OR (95% CI)	P-value
Age (per 1-year increase)	1.05 (1.04–1.05)	< 0.01	1.03 (1.02–1.04)	< 0.01
Male gender (vs. female)	1.15 (1.07–1.23)	< 0.01	1.32 (1.16–1.50)	< 0.01
**Education period**				
10–12 years (vs. ≤ 9 years)	0.85 (0.76–0.95)	< 0.01	1.01 (0.84–1.22)	0.93
> 13 years (vs. ≤ 9 years)	0.87 (0.78–0.98)	0.03	1.15 (0.94–1.40)	0.17
Living alone	1.23 (1.08–1.40)	< 0.01	1.13 (0.93–1.37)	0.21
BMI (per 1 SD increase)	0.90 (0.87–0.93)	< 0.01	0.88 (0.79–0.98)	0.02
Abdominal circumference (per 1 SD increase)	0.92 (0.89–0.95)	< 0.01	0.97 (0.87–1.08)	0.58
Alcohol consumption	0.88 (0.82–0.94)	< 0.01	0.82 (0.72–0.92)	< 0.01
Current smoking	0.58 (0.52–0.65)	< 0.01	0.61 (0.51–0.73)	< 0.01
History of hypertension	1.08 (0.99–1.17)	0.07	0.93 (0.83–1.06)	0.27
History of diabetes	1.81 (1.59–2.06)	< 0.01	1.60 (1.32–1.94)	< 0.01
History of dyslipidemia	1.16 (1.05–1.28)	< 0.01	1.06 (0.92–1.21)	0.44
History of cancer	1.23 (1.06–1.43)	< 0.01	1.08 (0.86–1.35)	0.50
History of angina/myocardial infarction	1.05 (0.87–1.27)	0.60	0.90 (0.67–1.20)	0.47
History of stroke	1.32 (1.06–1.65)	0.01	1.13 (0.81–1.58)	0.47
Exercise moderate (vs. mild)	2.95 (2.63–3.32)	< 0.01	2.74 (2.39–3.14)	< 0.01
intense (vs. mild)	4.43 (3.94–4.99)	< 0.01	3.88 (3.38–4.47)	< 0.01

*OR* odds ratio, *CI* confidence interval, *BMI* body mass index, *SD* standard deviation.

In addition, because exercise habits may result from health intentions and action, we performed an analysis excluding exercise habits as an explanatory factor. However, the factors associated with health intentions and behaviours were almost the same (“Supplementary Table”).

## Discussion

This study examined the factors associated with health intentions and behaviours among health checkup participants. The factors associated with health intentions were being young, being female, having a longer duration of education, having a high BMI and abdominal circumference, less daily exercise, diabetes, and dyslipidaemia. In contrast, the factors associated with health behaviours were being older, being male, having a low BMI, no alcohol consumption, no smoking, daily exercise, and diabetes. These results indicate that various factors have different associations with health intentions and behaviours in Japanese community residents.

Health and health behaviours are influenced at multiple levels, including the personal (i.e., biological, psychological), organisational/institutional, environmental (i.e., social and physical), and policy levels ^26^. Previous international studies reported that health behaviour-promoting factors include older age ^27^, female gender ^28^, urban residence ^29^, family structure, ethnicity ^30^, disease knowledge, higher education and health literacy ^31^, higher socioeconomic status ^32^ and non-depressive status ^33^. In Japan, Oda et al. reported that enhancing the “three pillars of health” (nutrition, exercise, and rest) is essential for health consciousness in older Japanese individuals ^34^. However, the individual factors related to health intentions and behaviours have not been determined. The current study clarified that being older, being female, and having a longer duration of education were associated with health intentions and behaviours in the Japanese population, similar to previous reports in other ethnic populations. In particular, young participants and females were likely to have health intentions but not health behaviours. In contrast, older people and males tended to be polarised into two groups: the no-intention group, and the action group.

According to a Japanese government survey of the general population, the proportion of respondents who reported having diabetes, dyslipidaemia, and hypertension as diseases of concern was comparable ^35^. In the current study, diabetes was significantly associated with both health intentions and behaviours. Dyslipidaemia was associated with health intentions but not with health behaviours, and hypertension and other comorbidities, including cancer, cardiovascular disease, and hypertension, were not associated with health intentions or behaviours. The reasons for this discrepancy are unclear. However, this finding indicates that obesity and diabetes were more strongly associated with health intentions and behaviours than other diseases.

The current findings have various clinical implications. For young females who exhibit health intentions but not health behaviours, it may be useful to provide opportunities to perform health behaviours. In contrast, for older males, who are less likely to have health intentions, it may be useful to provide guidance to foster health awareness in an easy-to-understand manner. In addition, individuals with dyslipidaemia and diabetes exhibited a higher frequency of health intentions and behaviours; therefore, they may represent a preferred target for health-promoting campaigns.

The strengths of the current study include the large sample size and range of factors considered, which increases the robustness of the findings. However, several limitations should be noted. First, there might have been selection bias in the occupation and age groups. The study participants were 40–74 years old covered by national health insurance, most of whom were self-employed, part-time workers, retirees, or unemployed. Therefore, other age groups (< 40 and ≥ 75 years old) and occupation groups (e.g., company workers) were not included. Data from a broader range of participants, including them, is necessary to verify our findings. However, each company usually conducts health checkups for these subjects, and there is no available database for them collectively. In addition, all study participants were Japanese. Therefore, the results of the current study may not apply to other ethnicities. Second, the causal relationships between health intentions, health behaviours, and associated factors could not be determined because of the nature of the cross-sectional study. To clarify the causal relationship between them, a Mendelian randomization analysis based on genomic and environmental exposure data in a prospective longitudinal study would be helpful to examines how these associated environmental factors affect the change of health intentions and behaviours status ^36–39^. Third, although this study included various explanatory factors, the information on other previously reported factors such as disease knowledge, health literacy ^31^, socioeconomic status ^32^, and depressive status ^33^ were not obtained. Therefore, there may be important factors associated with health intentions and behaviours that were not measured. Fourth, the question regarding health intentions and behaviours used in the current study was based on the five stages of the transtheoretical model of health behaviour change ^40^. Because health intentions and behaviours involve many aspects, including smoking, dietary habit, coping with stress, and physical inactivity ^3^, the assessment on each aspect is desirable. However, due to the capacity of the questionnaire, we used a single comprehensive question. It might provide only partial insight.

## Conclusions

This study revealed that health intentions and behaviours have different associations with several background factors in Japanese residents. Specifically, young females were more likely to have health intentions but not health behaviours, and older males were less likely to have health intentions. The results suggested that the presence of dyslipidaemia and diabetes were motivators to change health habits. The duration of education influenced participants’ health intentions. To conduct effective health-promotion campaigns, the characteristics of the intended audience should be considered.

## Methods

The Yamagata study aimed to clarify the relationships between genetic and environmental factors and common diseases and deaths using data from specified health checkup participants in seven cities in Yamagata Prefecture (Yamagata, Tendo, Kaminoyama, Sakata, Higashine, Sagae, and Yonezawa). These cities are small to medium-sized cities (population approximately 30,000–250,000) located in rural areas in the northern part of Japan. The study targeted local inhabitants covered by national health insurance, most of whom were agricultural workers, forestry workers, fisheries workers, self-employed, part-time workers, retirees, or unemployed. The details of this study have been reported in a previous study ^41^. The eligibility criteria selected participants of local health checkups from 2009 to 2015 who were 40–74 years old. The number of potential participants in this study was 28,528, and a total of 21,300 participants provided written informed consent to participate in the baseline survey of the Yamagata study. Of the 20,382 participants who filled out the questionnaire, 227 participants with missing answers in health intentions and behaviours and essential clinical information, including smoking, alcohol consumption, and medication, were excluded. The remaining 20,155 participants (8465 males and 11,690 females) were included in the final analysis of this study. Laboratory parameters were obtained at the health checkup site. A self-administered questionnaire was distributed to the study participants at the health checkup site and returned by post.

Information regarding age, gender, education period, the number of cohabitants, BMI, abdominal circumference, alcohol consumption, smoking habits, and history of hypertension, diabetes, dyslipidaemia, cancer, angina/myocardial infarction, and stroke were collected at baseline. Habitual alcohol consumption was defined as drinking alcohol every day or sometimes. Furthermore, information regarding the status of health intentions and behaviours was collected. The question used to collect this data was, “Do you want to improve your life habits of eating and exercising?” Five possible responses were provided for this question: (1) Do not want, (2) Do want to improve within 6 months, (3) Want to improve within a month, (4) Already trying to improve (< 6 months), and (5) Already trying to improve (> 6 months). This question is based on the five stages of the transtheoretical model of health behaviour change proposed by Prochaska et al. ^40^: (1: precontemplation, 2: contemplation, 3: preparation, 4: action, and 5: maintenance). Participants were categorised on the basis of their responses. Those who answered (1) were included in the “no intention” group, those who answered (2) or (3) were included in the “intention” group, and those who answered (4) or (5) were included in the “action” group.

Additionally, the SJ values, which quantify a portion of the amount of exercise per month, were collected for each individual. In the responses to the questions on strolling and jogging, we multiplied “frequency (per month)”, “time (per time)”, and “amount of exercise” to arrive at the SJ value ^42^. The SJ values were categorised in order of increasing value into three tertile groups: “mild”, “moderate”, and “intense”.

### Ethical considerations

This study was conducted in accordance with the Declaration of Helsinki and was approved by the Research Ethics Committee of the Yamagata University Faculty of Medicine (2019-390). The following ethical considerations were taken into account while conducting the study: the purpose and methods of the study, the free will to participate or withdraw from the study, the anonymity of the data, and the protection of privacy. These considerations were explained verbally and in writing. Written consent was obtained from all participants at the time of the baseline survey.

### Statistical analysis

The background factors of the individuals in the three groups (no intention, intention, and action) were examined using Fisher’s exact test for nominal variables. One-way analysis of variance was used for continuous variables. Next, we conducted a simple and multivariate logistic regression analysis of the factors associated with the intention and action. The intention group was compared with the no intention group; the action group were compared with the intention group. The explanatory factors identified were age, gender, education period, living alone, BMI, abdominal circumference, alcohol consumption, smoking, exercise habits, history of hypertension, diabetes, dyslipidaemia, cancer, angina/myocardial infarction, stroke, and SJ values. In addition, because exercise habits may result from health intention and action, we performed an analysis excluding exercise habits as an explanatory factor. The R statistical software package was used for data analysis, and the null hypothesis was rejected as false if the P-value was less than 0.05.

### Ethics approval and consent to participate

This study was approved by the Ethics Review Committee of the Faculty of the Medical Department of Yamagata University (approval number 2019-390). All participants provided written informed consent for participation.

## Supplementary Information

Supplementary Tables.

## Data Availability

The dataset of the current study is not publicly available for ethical reasons. However, upon reasonable request, it can be accessed by contacting the corresponding author.
