# Supplementary material for: Factors associated with health intentions and behaviour among health checkup participants in Japan
Source: Sci Rep. 2021 Oct 5;11:19761. doi: 10.1038/s41598-021-99303-y (PMC8492688; doi:10.1038/s41598-021-99303-y)
Supplement: Supplementary file 1 — Supplementary Tables. [file 41598_2021_99303_MOESM1_ESM.docx]

|  | Intention vs. No intention | |  | Action vs. Intention | |
| --- | --- | --- | --- | --- | --- |
|  | OR (95%CI) | P-value |  | OR (95%CI) | P-value |
| Age (per 1-year increase) | 0.96 (0.96-0.97) | < 0.01 |  | 1.05 (1.04-1.05) | < 0.01 |
| Male gender (vs. Female) | 0.58 (0.52-0.64) | < 0.01 |  | 1.34 (1.20-1.49) | < 0.01 |
| Education period 10-12 years (vs. < 9 years) | 1.38 (1.21-1.57) | < 0.01 |  | 0.98 (0.85-1.14) | 0.79 |
| > 13 years (vs. < 9 years) | 1.66 (1.44-1.92) | < 0.01 |  | 1.14 (0.97-1.33) | 0.11 |
| Living alone | 1.23 (1.05-1.44) | 0.01 |  | 1.13 (0.97-1.32) | 0.11 |
| BMI (per 1 SD increase) | 1.11 (1.02-1.21) | 0.01 |  | 0.94 (0.86-1.02) | 0.16 |
| Abdominal circumference (per 1 SD increase) | 1.32 (1.22-1.44) | < 0.01 |  | 0.89 (0.81-0.97) | < 0.01 |
| Alcohol consumption | 1.06 (0.96-1.16) | 0.23 |  | 0.86 (0.78-0.95) | < 0.01 |
| Current smoking | 0.98 (0.86-1.11) | 0.70 |  | 0.60 (0.51-0.69) | < 0.01 |
| History of hypertension | 1.11 (1.00-1.22) | 0.04 |  | 0.88 (0.79-0.97) | 0.01 |
| History of diabetes | 1.42 (1.18-1.71) | < 0.01 |  | 1.61 (1.38-1.89) | < 0.01 |
| History of dyslipidemia | 1.53 (1.35-1.73) | < 0.01 |  | 1.06 (0.95-1.19) | 0.32 |
| History of cancer | 1.19 (0.99-1.43) | 0.07 |  | 0.98 (0.82-1.18) | 0.85 |
| History of angina/myocardial infarction | 1.05 (0.83-1.32) | 0.69 |  | 0.88 (0.69-1.11) | 0.28 |
| History of stroke | 1.07 (0.81-1.42) | 0.63 |  | 1.10 (0.84-1.44) | 0.51 |
| OR: odds ratio, CI: confidence interval, BMI: body mass index, SD: standard deviation. | | | | | |

Supplementary Table. Factors associated with Intention to change and Action: Multivariate logistic regression analysis (excluding Exercise).
